# Nanopore basecalling from a perspective of instance segmentation

**DOI:** 10.1186/s12859-020-3459-0

**Published:** 2020-04-23

**Authors:** Yao-zhong Zhang, Arda Akdemir, Georg Tremmel, Seiya Imoto, Satoru Miyano, Tetsuo Shibuya, Rui Yamaguchi

**Affiliations:** 1 The Institute of Medical Science, The University of Tokyo, Shirokanedai 4-6-1, Minato-ku, Tokyo, 108-8639 Japan; 2 Division of Cancer Systems Biology, Aichi Cancer Center Research Institute, 1-1 Kanokoden, Chikusa-ku, Aichi, Nagoya, 464-8681 Japan; 3 Division of Cancer Informatics, Nagoya University Graduate School of Medicine, 65 Tsurumai-cho, Showa-ku, Aichi, Nagoya, 466-8550 Japan

**Keywords:** Nanopore basecalling, Deep learning, UR-net

## Abstract

**Background:**

Nanopore sequencing is a rapidly developing third-generation sequencing technology, which can generate long nucleotide reads of molecules within a portable device in real-time. Through detecting the change of ion currency signals during a DNA/RNA fragment’s pass through a nanopore, genotypes are determined. Currently, the accuracy of nanopore basecalling has a higher error rate than the basecalling of short-read sequencing. Through utilizing deep neural networks, the-state-of-the art nanopore basecallers achieve basecalling accuracy in a range from 85% to 95%.

**Result:**

In this work, we proposed a novel basecalling approach from a perspective of instance segmentation. Different from previous approaches of doing typical sequence labeling, we formulated the basecalling problem as a multi-label segmentation task. Meanwhile, we proposed a refined U-net model which we call UR-net that can model sequential dependencies for a one-dimensional segmentation task. The experiment results show that the proposed basecaller URnano achieves competitive results on the in-species data, compared to the recently proposed CTC-featured basecallers.

**Conclusion:**

Our results show that formulating the basecalling problem as a one-dimensional segmentation task is a promising approach, which does basecalling and segmentation jointly.

## Background

Nanopore sequencing, a third-generation sequencing technique, has achieved impressive improvements in the past several years [1, 2]. A nanopore sequencer measures currency changes during the transit of a DNA or an RNA molecule through a nanoscopic pore and can be equipped in a portable size. For example, MinION is such a commercially available device produced by Oxford Nanopore Technologies (ONT). One key merit of nanopore sequencing is its ability to generate long reads on the order of tens of thousands of nucleotides. Besides the sequencing application, it is actively used in more and more fields, such as microbiology and agriculture.

Basecalling is usually the initial step to analyze nanopore sequencing signals. A basecaller translates raw signals (referred to as squiggle) into nucleotide sequences and feeds the nucleotide sequences to downstream analysis. It is not a trivial task, as the currency signals are highly complex and have long dependencies. ONT provides established packages, such as Scrappie and Guppy. Currently, nanopore basecalling still has a higher error rate when compared with short-read sequencing. Its error rate ranges from 5% to 15%, while the Illumina Hiseq platform has an error rate of around 0.1% (a majority of reads have Q-score more than 30). More and more work is now focusing on solving challenges to further improve basecalling accuracy.

Early-generation basecallers require first splitting raw signals into event segments and predict k-mer (including blanks) for each event. Sequential labeling models, such as hidden Markov model (HMM) [3] and recurrent neural network (RNN) [4] are used for modeling label dependencies and predicting nucleotide labels. It is widely considered that a two-stage pipeline usually brings about an error propagation issue that wrong segments affect the accuracy of basecalling. Recently, end-to-end deep learning models are used to avoid pre-segmentation of raw signals, which enables basecallers to directly process raw signals. For example, BasecRAWller [5] puts the event segmentation step in a later stage after initial feature extraction by an RNN. Chiron [6] and recent ONT Guppy use a Connectionist Temporal Classification (CTC) module to avoid explicit segmentation for basecalling from raw signals. With CTC, a variant length base sequence can be generated for a fixed-length signal window through output-space searching.

On the other hand, even though those basecallers can translate raw signals to bases directly, segmentation and explicit correspondence between squiggles and nucleotide bases are also informative. It can provide information for detecting signal patterns of target events, such as DNA modifications [7]. In a re-squiggle algorithm, basecalling and event detection are also required.

In this paper, we do basecalling from the point of view of instance segmentation and develop a new basecaller named URnano. Distinguished from previous work that treats basecalling as a sequence labeling task, we formalize it as a multi-label segmentation task that splits raw signals and assigns corresponding labels. Meanwhile, we avoid making the assumption that each segment is associated with a k-mer (*k*≥2) and directly assign nucleotide masks for each currency sampling point. On the model-level, based on the basic U-net model [8], we propose an enhanced model called UR-net that is capable of modeling sequential dependencies for a one-dimensional (1D) segmentation task. Our basecaller is also an end-to-end model that can directly process raw signals. Our experiment results show that the proposed URnano achieves competitive results when compared with current basecallers using CTC decoding.

## Methods

**Fig. 1:**
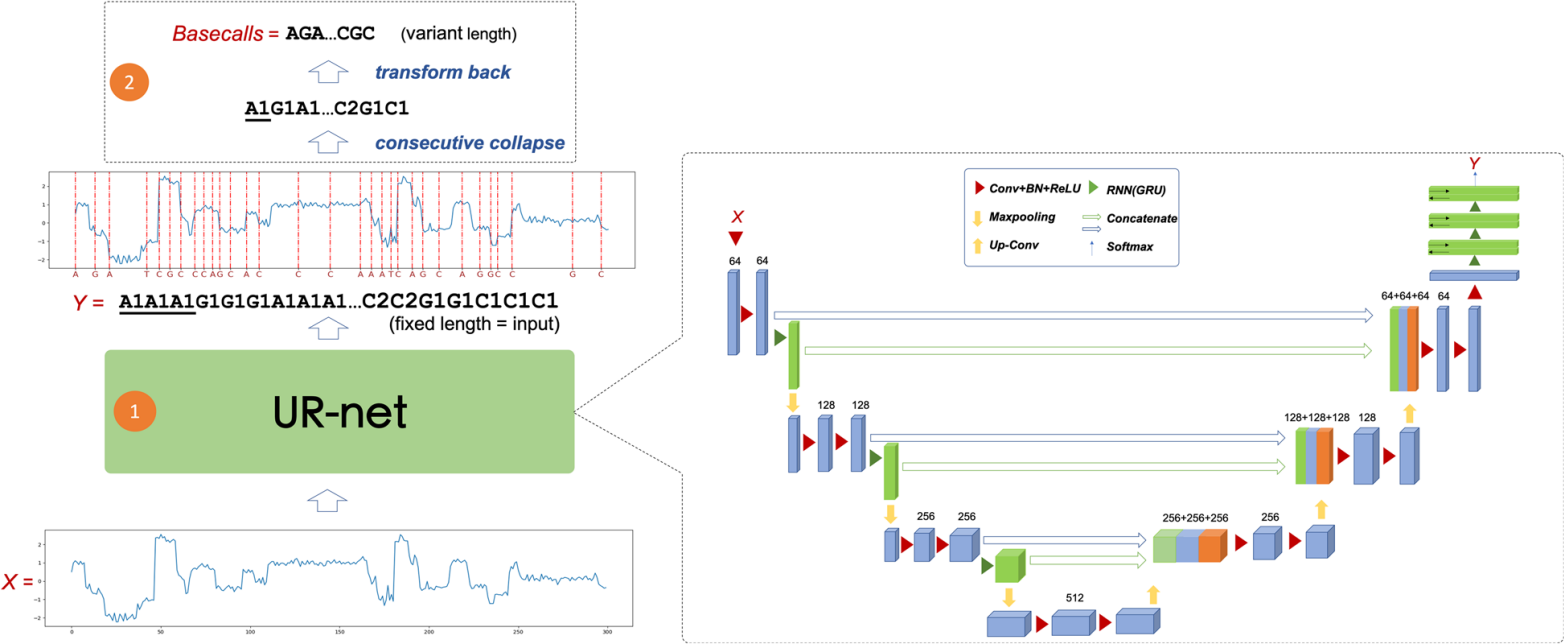
Overall pipeline of URnano basecaller. Block ① is the UR-net deep neural network. Block ② is the post-processing part that transforms the UR-net’s output to final basecalls

The overall pipeline of URnano is described in Fig. 1. URnano contains two major components: ① UR-net for signal segmentation and basecalling. ② Post-processing. For streaming signals generated by a nanopore sequencer, URnano scans signals in a fixed window length *L* (e.g., *L*=300) and slides consequently with a step length *s* (e.g., *s*=290). Given signal input *X*=(*x*_1_,*x*_2_,...,*x*_*i*_,...,*x*_*L*_), UR-net predicts segment label masks *y*_*i*_ for each *x*_*i*_. The output of UR-net *Y*=(*y*_1_,*y*_2_,...,*y*_*i*_,...,*y*_*L*_) has exactly the same length as the input *X* and *y*_*i*_∈{*A*_1_,*A*_2_,*C*_1_,*C*_2_,*G*_1_,*G*_2_,*T*_1_,*T*_2_}. Here, {*A*_1_,*C*_1_,*G*_1_,*T*_1_} and {*A*_2_,*C*_2_,*G*_2_,*T*_2_} are alias label names, which is designed to handle homopolymer repeats (described in “Homopolymer repeats processing” section). After label mask *Y* is generated, we conduct a post-processing step that transforms *Y* to *Y*^′^∈{*A*,*C*,*G*,*T*}^*N*^, where *N* is the length of the final basecall. The post-processing contains two simple steps. First, it collapses consecutive identical label masks as one label. Second, the collapsed labels in alias namespace are transformed back to bases in {*A*,*C*,*G*,*T*}. *Y*^′^ is the final basecalls of the URnano.

Besides predicting basecalls, URnano also generates a signal segment for each base. In the previous work [4, 5], signal segments are assumed to be associated with k-mers of a fixed k (e.g., k=2,4,5). Every base is read as a part of k consecutive events. In URnano, we avoid making the k-mer assumption and directly assign label masks for signals.

### UR-net: enhanced u-net model for 1D sequence segmentation

The key component of the URnano is UR-net. Its network structure is profiled in Fig. 1 (more details in Additional file 1: Figure S1). **In general, UR-net is based on the U-net model [**8**] and is enhanced to model sequential dependencies**. “R" represents a refinement of U-net and the integration of RNN modules. The original U-net is designed for image data in two dimensional (2D) and has achieved the-state-of-the-art performances in many image segmentation tasks. Although the model can be directly applied for 1D data, the 1D segmentation task has its own characteristics that are distinguished from the 2D image segmentation task. In a sequence segmentation task, one segment may not only relate to its adjacent segments but also depends on non-adjacent segments that are several distance away. Such dependencies were not considered in the original U-net model, which mainly focuses on detecting object regions and boundaries.

The UR-net has a similar U-shape structure as U-net, in which left-U side encodes inputs *X* through convolution (CONV) with batch normalization (BN) following with rectified linear unit (ReLU) and max pooling, and right-U side decodes through up-sampling or de-convolution. We make two major enhancements in the UR-net model, which are highlighted in green shown in Fig. 1 and described as follows:

- For the encoding part (left-U), we add an RNN layer right after each CONV-BN-ReLU block to model sequential dependencies of hidden variables in different hierarchical levels. Those RNN layers are also concatenated with UP-Sample layer in the right-U decoding part.
- We add three bi-directional RNN layers as final layers.

Those changes are motivated to enhance the sequential modeling ability of the U-net.

### Model training

Given *D*={(*X*_*i*_,*Y*_*i*_)|*i*=1,...,*n*}, we train UR-net with an interpolated loss function that combines dice loss (DL) and categorical entropy loss (CE). Note that the task loss of edit distance can not be directly optimized. For each segment sample *i*, *DL*_*i*_ and *CE*_*i*_ are defined as follows:

1 $$\begin{array}{*{20}l} DL_{i} &= \frac{2\sum_{t=1}^{L}\sum_{j=1}^{8}p_{t,j} \times g_{t,j}} {\sum_{t=1}^{L}\sum_{j=1}^{8}g_{t,j} + \sum_{t=1}^{L}\sum_{j=1}^{8}p_{t,j}} \end{array} $$

2 $$\begin{array}{*{20}l} CE_{i} &= \sum_{t=1}^{L}\sum_{j=1}^{8} g_{t,j} \times log(p_{t,j}) \end{array} $$

where *t*={1,...,*L*} represents the *t*-th time step in the sequence. For each time step *t*, we do one-hot encoding for prediction label *p*_*t*_ and gold label *g*_*t*_ in the 8-label space of {*A*_1_,*A*_2_,*T*_1_,*T*_2_,*G*_1_,*G*_2_,*C*_1_,*C*_2_}. *p*_*t*,*j*_ is the softmax value for *j*-th label in time step *t*. *g*_*t*,*j*_∈{0,1} indicates the gold label in time step *t*.

We interpolate the dice loss and the categorical entropy loss with weight *α* and *β*.

3 $$\begin{array}{@{}rcl@{}} loss &=& \alpha \sum_{i=1}^{n} CE_{i} + \beta \sum_{i=1}^{n} DL_{i} \end{array} $$

By default, *α*=*β*=1. We use Adam [9] to optimize the above loss function.

### Homopolymer repeats processing

**Fig. 2:**
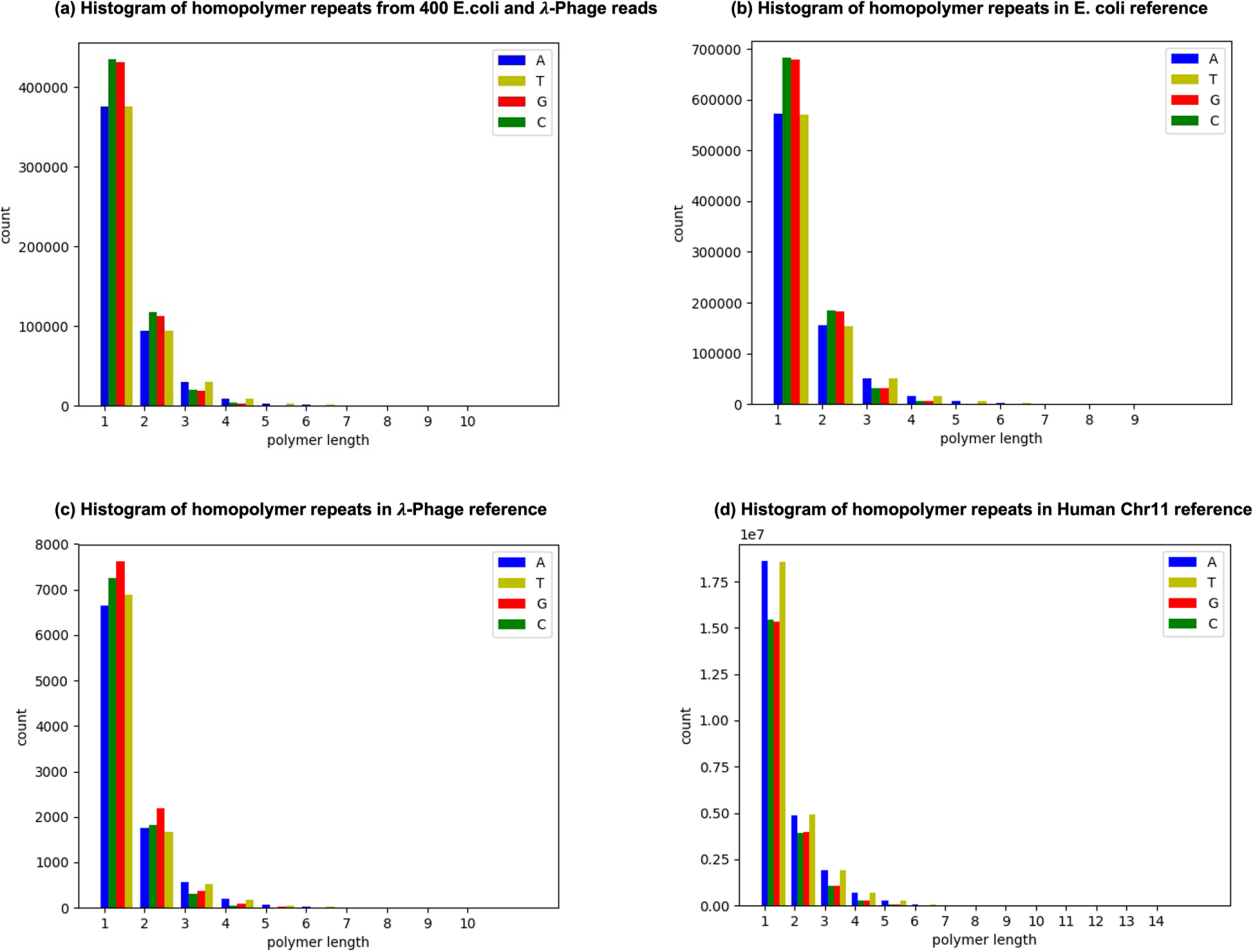
**a** is the histogram of homopolymer repeats from 400 E. coli and *λ*-phage reads. **b**, **c** and**d** are histograms of homopolymer repeats for real E.coli, *λ*-phage and Human Chr11 reference genome. Here, the single nucleotide is also treated as a “homopolymer" for reference

In genomes, homopolymer repeats (e.g. AAA and TTTT) commonly exist. Figure 2a demonstrates a histogram of homopolymer repeats on randomly sampled 200 E. coli reads and 200 *λ*-phage reads. From the figure, we can observe that majority homopolymer repeats have lengths less than 5 base-pairs. Figure 2b-d are homopolymer repeats on reference genomes. For the original U-net model, adjacent bases in a homoploymer can not be distinguished and are merged as one base. This brings about deletion errors if models are directly trained on this data. To solve this problem, we use an alias trick to differentiate adjacent identical labels. For example, homopolymer repeat “*AAAAA*" in the training data is converted to “ *A*_**1**_***A***_**2**_***A***_**1**_***A***_**2**_***A***_**1**_" for training UR-net model. In the inference stage, those new labels are transformed into the original representation through post-processing.

### Merge basecalls in sliding window into a whole read

In the training phase, a read is split into the non-overlapping windows of fixed length. In the testing phase, for calculating read accuracy, read signals are scanned with overlapping windows. The sliding window takes a step *s* (*s*<*L*). For each time step *t*, *x*_***t***_** and ***x*_***t***−1_** have ***L*−*s* overlaps on the signal content. Thus, for a read signal of length *N* we have $\lfloor \frac {N-L}{s} \rfloor $ windows and each overlap with its neighbors by *L*−*s*. The basecalls for each input at neighboring positions are merged in pair-wise fashion consecutively.

**Fig. 3:**
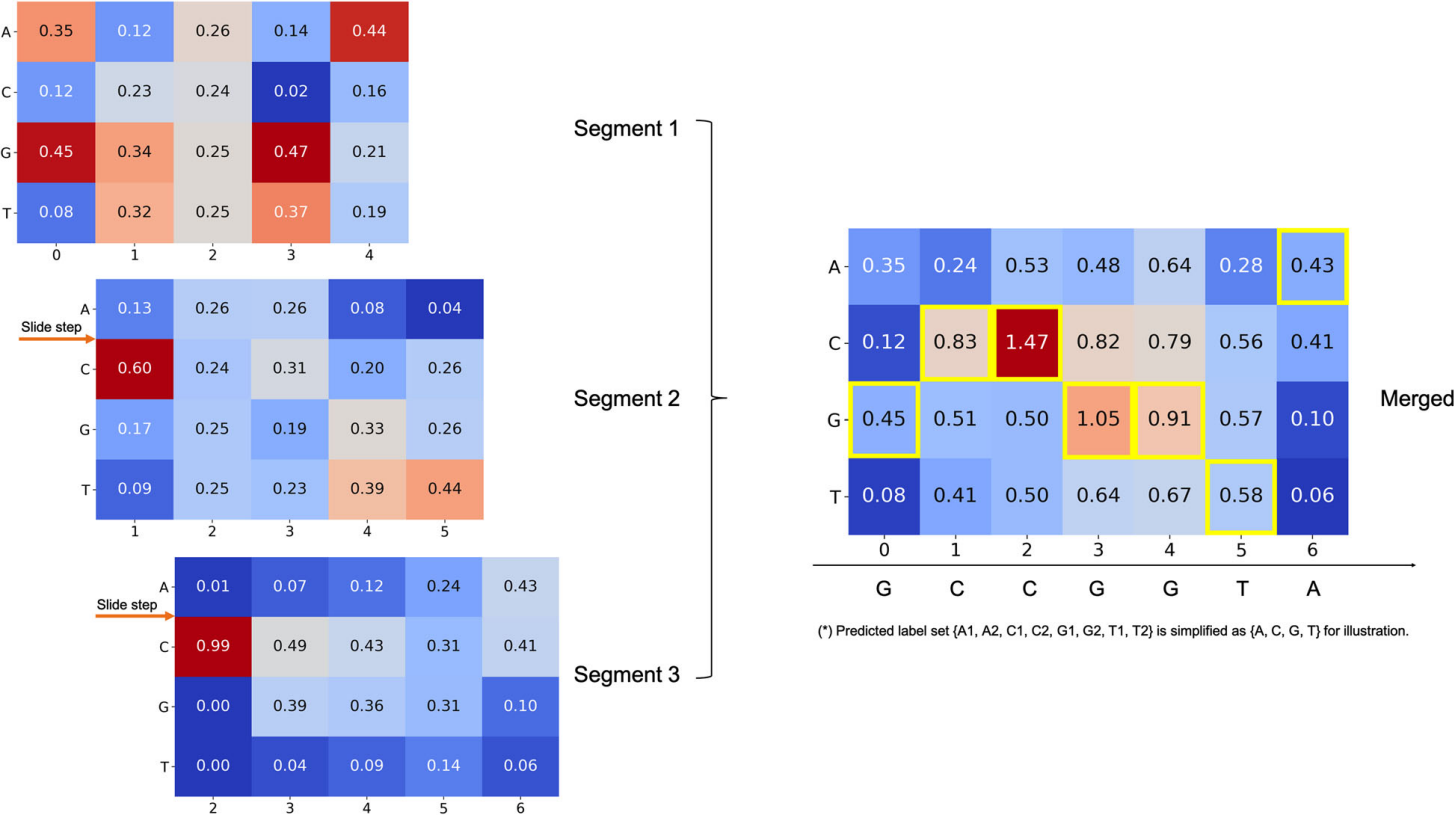
An example on merging basecalls of overlapped slide window using soft merging

To merge the basecalls of sliding windows, we have two different strategies in general. One is on the nucleotide level after the final basecall is generated. The other is on the segment label level before the final basecalls. Here, we use the latter strategy with ‘soft merging’. Shown in Fig. 3, the soft merging combines consecutive predictions at the segment label level, where we use probabilities of each segment label predicted by the deep learning model. We apply weight interpolation for each overlapped position and use the label mask with the maximum score as the prediction label for the overlapped positions. The basecalls are made after merging all sliding windows of a read.

### Experiment settings

**Data**: we compared URnano with the latest version of related basecallers: Chiron (v0.5.1) and ONT Guppy (v3.2.2). Both Chiron and Guppy use CTC decoding for basecalling. For comparing model performances, we used a publicly accessible curated dataset provided by Teng et al. [6]. The dataset contains per-base nucleotide labels for currency segments. In other words, we know the signal segment for each nucleotide. The training set contains a mixture of randomly selected 2000 E. coli reads and 2000 *λ*-phage reads generated using nanopore’s 1D protocol on R9.4 flowcells. The test set contains the same amount of reads from E. coli and *λ*-phage. To assess read accuracy and assembly performance across species, we use 1000 randomly selected reads from Chromosome 11 (Chr11) of human benchmark sample NA12878 (1D protocol on R9.4 flowcells).

The raw signals are normalized using median shift and median absolute deviation scale parameters $Norm\_signal = \frac {Raw\_signal - Shift}{Scale}$. The *Norm*_*signal* usually has values in a range of [−2,2]. For training deep learning models, signals of a read are split into non-overlapping window segments of a fixed length *L* (*L*=300 by default). For those samples containing *Norm*_*signal* larger than 10, we filtered them out for training. In total, we have 830,796 segments of 300-length used for training.

**Evaluation metric**: we evaluated a basecaller’s performance according to the following metrics:

- Normalized edit distance (NED) between gold nucleotides and basecalls in non-overlapping windows. It is used to evaluate different deep learning models.
- Read accuracy (RA) evaluates the difference between a whole read and its reference
  $$ RA = \frac{M}{ M + U + I + D} \quad. $$
- Read identity rate (RI)
  $$ RI = \frac{M}{\text{number of bases in reference}} \quad, $$
  where *M* is the number of bases identical to the reference. *U*, *I* and *D* are the numbers of mismatches, inserts, and deletions, respectively, according to the reference read. Following the evaluation scheme in Chiron, we used GraphMap (v0.5.2) [10] to align basecalls of a read to the reference genome. The error rates of the aligned reads are calculated using the publicly available Japsa tool (v1.9-3c).
- Assembly identity (AI) and relative length (RL). We assembled genomes using the results of each basecaller. Assembly identity and relative length are calculated by taking the mean of individual accuracy rates and relative lengths for each shredded contig, respectively. The details of the assembling process are described in “Read assembly results” section.
  $$ AI = \frac{1}{N}\sum_{i=1}^{N} RA_{i} \quad, \quad RL = \frac{1}{N}\sum_{i=1}^{N}\frac{L_{pred_{i}}}{L_{ref_{i}}}\quad, $$
  where *N* is the total number of aligned parts, $L_{pred_{i}}$ is the length of the assembled *i*^***t****h*^** basecall and** *L*_***ref***_ is the length of the reference genome.

**Model and basecaller settings**: The URnano is implemented using Keras (v2.2.4) with Tensorflow backend (v1.8.0). We trained three basecallers on the same dataset with input sequence length of 300. The Chiron decodes with its default beam size of 50. Guppy is trained with ONT Taiyaki (v4.1.0) with default setting.

## Results

### Basecalling results on non-overlapping segments

We first investigated different deep network architectures in the URnano framework using normalized edit distance (NED). In total, 847,201 samples of 300-length window are evaluated. In general, the lower the NED is, the more accurate a basecaller is.

Table 1 shows NED of using different neural network architectures. The original U-net performs the worst of 0.3528, while UR-net achieves the best of 0.1665. As the sequential dependencies are not modeled in the U-net, these results indicate the importance of sequential information in the 1D segmentation task for basecalling.

**Table 1:** NED of URnano with different network architectures for the non-overlapping window in the test set

**Network structure**	**Mean**	**Std**
U-net	0.3528	0.2448
3GRU	0.2808	0.1631
U-net+3GRU	0.1800	0.1296
UR-net	**0.1665**	0.1329

To take into account the sequential dependencies, we initially added 3 layers of bi-directional gated recurrent units (GRU) for the output of the U-net. This gives about 0.1728 absolute reduction on the NED compared with the U-net. Meanwhile, we observed that the U-net+3GRU performs significantly better than only using 3GRU (0.1 absolute NED reduction). In addition, we incorporated GRU layers in different hierarchical levels of convolutional layers. It gives a further 7.5% relative reduction of NED, when comparing URnano with U-net+3GRU.

### Basecalling results on read accuracy

We evaluated read accuracy for the whole reads on the test set. The results are summarized in Table 2. We first investigated in-species evaluation where the training data contains data of the same species as the test set. We tested on 2000 E. coli and 2000 *λ*-phage reads, separately. For E.coli, Guppy _taiyaki achieves the best RA score of 0.8636, while URnano has the highest RI of 0.9010. They all perform significantly better than Chiron. For *λ*-phage, URnano performs better on both RA and RI than the other two basecallers. But the performance gap in RA between URnano and Guppy _taiyaki is not large. For cross-species evaluation, we evaluated on human data by doing basecalling on 1000 randomly selected reads from Chr11. Compared with the evaluation of in-species, the performances of all three basecallers are decreased. From Fig. 2, an obvious difference of GC-content between E. coli/ *λ*-phage and human can be observed. Such a difference between training and test brings about a performance drop for deep-learning-based basecallers. Guppy_taiyaki performs best among all three basecallers on the human data, which is around 0.015 higher on RA and 0.011 higher on RI than URnano. In all three species, URnano achieves the lowest mismatch rate.

**Table 2:** Results of read accuracy on the test set

**Species**	basecaller	**Deletion**	**Insertion**	**Mismatch**	**Read Identity**	**unaligned**	**Read Accuracy**
E. coli	Chiron	0.0692	0.0465	0.0600	0.8709	7/2000	0.8243
	URnano	**0.0584**	0.0533	**0.0407**	**0.9010**	8/2000	0.8476
	Guppy _taiyaki	0.0585	**0.0343**	0.0436	0.8978	7/2000	**0.8636**
*λ*-phage	Chiron	0.0799	0.0467	0.0641	0.8559	9/2000	0.8093
	URnano	0.0662	0.0455	**0.0363**	**0.8975**	10/2000	**0.852**
	Guppy _taiyaki	**0.0655**	**0.0397**	0.0481	0.8864	6/2000	0.8467
Human	Chiron	0.0983	**0.0687**	0.0866	0.8151	385/1000	0.7464
	URnano	0.0957	0.0788	**0.0727**	0.8316	375/1000	0.7528
	Guppy _taiyaki	**0.0822**	0.0748	0.0756	**0.8422**	352/1000	**0.7674**

### Read assembly results

We also evaluated the quality of the assembled genomes using the reads generated by each basecaller on the test set. We make use of the same evaluation pipeline of Teng et al. [6]. Assembly experiments consist of three steps: read mapping, contig generation, and polishing. Read mapping uses *minimap2* (v2.17-r943-dirty) [11], which is designed for mapping each long-read with high-error rate in a pairwise manner. After that, *miniasm* (v0.3-r179) is applied to generate long contigs based on the pairwise read alignment information generated in the previous read mapping phase. Finally, *Racon* (v1.4.6) [10] is used to polish the contigs by removing the read errors iteratively. The polishing step consists of mapping the initial long-reads to the contigs and takes the consensus of each mapped read to get higher quality contigs. Polishing is repeated 10 times.

In evaluating the quality of output contigs, each contig is shredded into 10k-base components and aligned to the reference genome. We evaluated the identity rate of each 10k-base component and report the mean of all the 10k-base components as the final identity rate of the assembly. The identity rate is the number of matching bases divided by the total length of the aligned part of the reference. This identity is also referred to as the ‘Blast identity’ [12]. If the total length of the aligned parts is smaller than half of the read length, we assume it to be unaligned and the identity rate for that contig is 0. Relative length is also calculated in a similar manner.

Table 3 gives the assembly results on E. coli, *λ*-phage and Human test sets (Polished assembly result of each round can be found in Additional file 2: Table S1). Note that different than the conventional approach which evaluates assembly results on relatively higher depth data, our test data is shallow, especially for human data. The read assembly here is mainly used as a side evaluation metric for basecalling. The reference genomes used for each species data are on different scales. *λ*-phage has the smallest size of 52k bp, while Chr11 has the largest size of 135M bp. E. coli has a number of around 4.6M bp in the middle. Under the circumstance of using a few reads for assembly, species with a smaller genome size tends to have more overlapped reads. On *λ*-phage data, URnano performs the best on both AI and RL.

**Table 3:** Assembly results on the test set

**Dataset**	basecaller	**Assembly Identity (%)**	**Relative Length (%)**
E. coli	Chiron	97.1995	99.0589
	URnano	97.4994	**99.8868**
	Guppy _taiyaki	**98.272**	99.3644
*λ*-phage	Chiron	90.8901	99.8352
	URnano	**99.6525**	**99.9211**
	Guppy _taiyaki	99.1734	99.3223
Human	Chiron	92.0374	**99.9770**
	URnano	91.2176	101.553
	Guppy _taiyaki	**93.2813**	99.7087

The mean values of 10-round polished results are reported

In the in-species evaluation, we observed a correlation between the assembly identity and the read accuracy, that a basecaller with a higher RA tends to have a higher AI (the Pearson’s correlation coefficient between AI and RA is 0.83). In the cross-species evaluation, the Pearson’s correlation reduces to 0.76. Guppy_taiyaki achieves the best AI with the highest RA of 93.28% on the human data. For the relative length (the closer RL to 100% the better), URnano performs the best on both E.coli and *λ*-phage data, but has a longer relative length on the cross-species data. It is consistent with the result shown in Table 2 that URnano has a higher insertion rate on the human data.

### Segmentation results

**Fig. 4:**
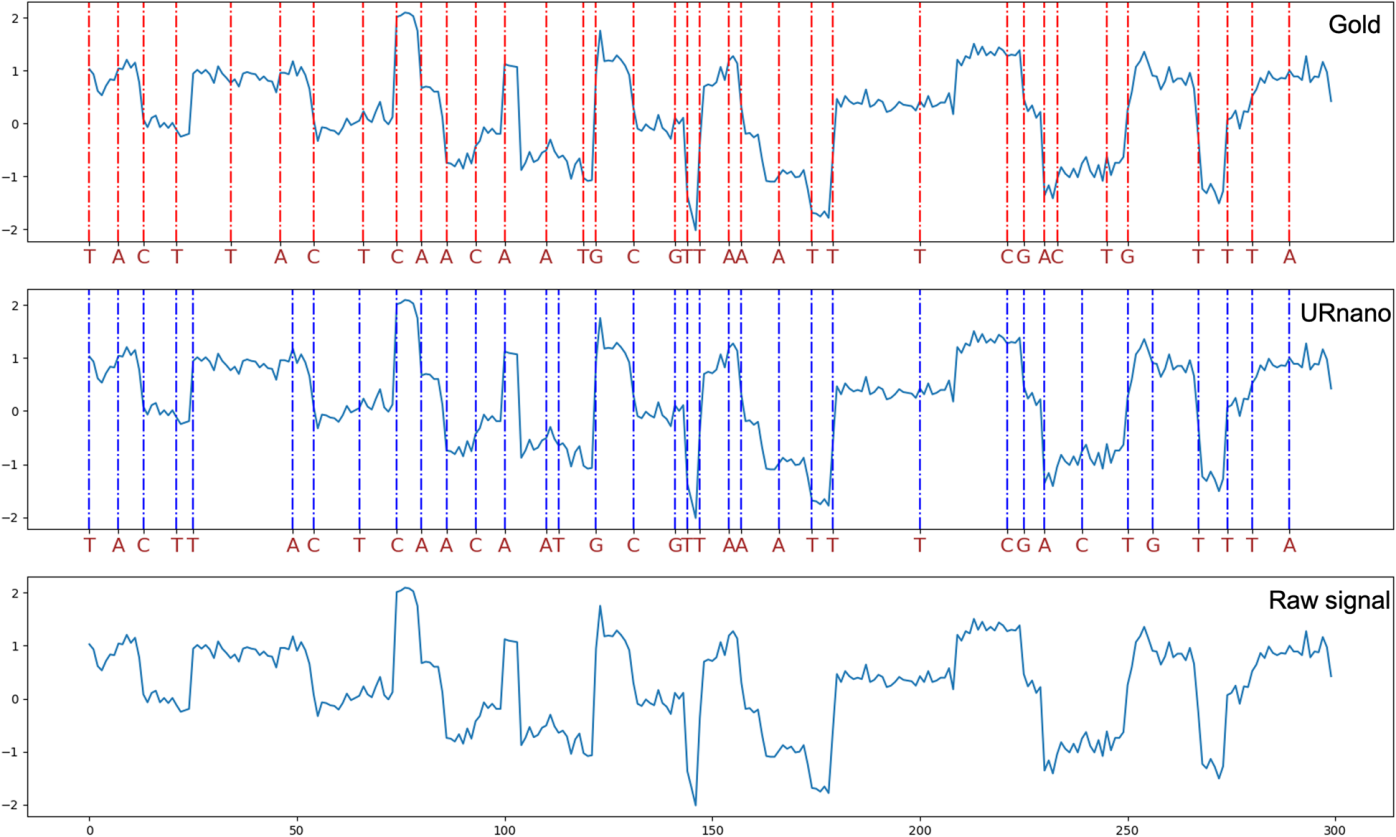
A segmentation example of the URnano basecall for the sequence of “TACTTACTCAACAATGCGTTAAATTTCGACTGTTTA”. A dotted vertical line indicates the start position of a nucleotide segment

In this section, we investigated event segments for each predicted nucleotide. Figure 4 demonstrates an example of basecalling and segmentation by URnano. For URnano, the signal segment for each base can be directly derived through label masks. As in the post-process of URnano, consecutive identical masks are merged as one base, a region of consecutive identical masks is just an event segment.

For CTC-based basecaller, segmentation is not explicitly conducted or learned in the model. Although heuristic approaches can be used to derive segments based on intermediate logit output, it is not straightforward and accurate to determine per-base segmentation using CTC basercallers. Figure 4 demonstrates the segmentation results generated by URnano for a randomly selected input. From the gold segmentation, we can observe **the length of signals for each nucleotide is not evenly distributed across time**. This is mainly due to the fact that, the speed at which a molecule passes through a pore changes over time. The speed issue makes the segmentation a non-trivial task. Traditional statistical approaches without considering the speed changes may not work. Here, the proposed URnano is designed to learn segmentation from the data, which implicitly considers the speed changes embedded in signals. For example, events of ‘T’s around 150 time-step tend to have short lengths than that in 200 time-step. The URnano can distinguish such speed changes as shown in the third row of the figure. For the beginning part of the signal in this example, URnano makes the correct base predictions, but the segments of ‘TT’ shift a bit compared to the gold standard.

### Speed comparison

We measured the speed of the basecallers by basepairs-per-second metric. To calculate the speed, we divided the total length of basecall by the total time. URnano achieves 16,271.15 bp/s on average, which is around 1.77x faster than Chiron with 9,194.78 bp/s on average using Nvidia Tesla V100 GPU under single thread setting. We used the Chiron’s script to generate basecalling speed for URnano and Chiron. Note that the previous version of Chiron (v0.3) is slow with using large overlapping of consecutive sequences (90%), while the latest version (v0.5.1) uses smaller overlap for speedup at the cost of certain read accuracy. Both URnano and Chiron were not optimized for speed as Guppy, which are 2-3 orders of magnitude slower than Guppy with a reported speed of ∼1,500,000 bp/s [12].

## Discussion

We analyzed the three basecallers and enumerated their key modules including network input, network structure, network output and post-process of each one, shown in Table 4. For neural network architectures, the CNN layer and RNN layer are commonly used. CNN is generally used to extract features from raw signals and prepares input for RNN. RNN module is used to learn dependencies among hidden units. With URnano, our experiment also demonstrates the usefulness of using RNN for 1D segment mask prediction. Besides using RNN in final layers, it also demonstrates the combination of CNN and RNN layers in the encoding stage can further improve the basecalling performance. Chiron and Guppy use CTC decoding to generate basecalls of variant length through beam-searching in the hidden unit space. The output of Chiron includes blank labels, which are collapsed in the CTC decoding stage.

**Table 4:** A brief summary of related deep-learning-based basecallers

**Module**	**Chiron**	**Guppy**	**URnano**
**Input**	Raw	Raw	Raw
**Networks**	CNN+RNN+CTC	RGRGR+CTC	UR-net
**Output**	bases	bases	base masks
**Post-process**	N/A	N/A	label transform

In a real physical process, the speed of a molecule passing through a nanopore changes over time. This can be observed in the Fig. 4. A k-mer assumption using fixed k may not hold over time. Although incorporating blank labels can deal with the low-speed case, the high-speed one that involves more bases for the same signal length could exceed the limit of the fixed k. For Chiron and URnano, the fixed k-mer assumption is avoided. Chiron uses CTC decoding, while URnano uses label masks that are smaller units than 1-mer.

To curate the data for training a basecaller, a re-squiggle algorithm is usually applied. In a re-squiggle algorithm, raw signal and associated basecalls are refined through alignment to a reference. After re-squiggling, a new assignment from squiggle to a reference sequence is defined. In the re-squiggle algorithm [7], event detection and sequence to signal assignment are performed separately. We think the proposed URnano can be used as the basecaller in a re-squiggle algorithm, as it can do basecalling, event detection and sequence to signal assignment jointly in an end-to-end manner. URnano can also be extended to detect DNA methylation, in which event segments are usually required.

In this paper, we only evaluated on a small curated data for fair comparisons between different basecallers. URnano works better on in-species evaluation. To further improve URnano, we intend to train it on larger data covering more species.

## Conclusion

In this paper, we proposed a novel basecalling approach from the perspective of instance segmentation. We formalized basecalling as a multi-label segmentation task and developed an end-to-end solution that can perform basecalling from raw signals and generate signal segments for basecalls at the same time. In addition, we proposed an enhanced deep neural network architecture called UR-net for 1D sequence data. The proposed URnano outperforms Chiron in both in-species and cross-species evaluation on read accuracy, and achieves competitive assembly results in the in-species evaluation. Although the performance of URnano (read accuracy and basecalling speed) still has a distance to the-state-of-the-art ONT Guppy, it provides an alternative basecalling approach that can generate per-base segmentation information jointly.

## Supplementary information

**Additional file 1** Figure S1. UR-net’s network structure plotted by Keras.

**Additional file 2** The polished assembly results of each round in the 10-round polishing process.

## Data Availability

The source code and trained model of URnano are available on Github (https://github.com/yaozhong/URnano). The training and testing data used in this work can be downloaded in GigaScience Database (http://dx.doi.org/10.5524/100425). Nanopore data of NA12878 is publicly available by nanopore wgs consortium (https://github.com/nanopore-wgs-consortium/NA12878).

## Abbreviations

CTC: Connectionist temporal classification
HMM: Hidden Markov model
CNN: Convolutional neural network
CONV: Convolution
BN: Batch normalization
ReLU: Rectified linear unit
RNN: Recurrent neural network
GRU: Gated recurrent unit
ONT: Oxford Nanopore technologies
