# Supplementary figures and images for: Nanopore basecalling from a perspective of instance segmentation
Source: BMC Bioinformatics. 2020 Apr 23;21(Suppl 3):136. doi: 10.1186/s12859-020-3459-0 (PMC7178565; doi:10.1186/s12859-020-3459-0)

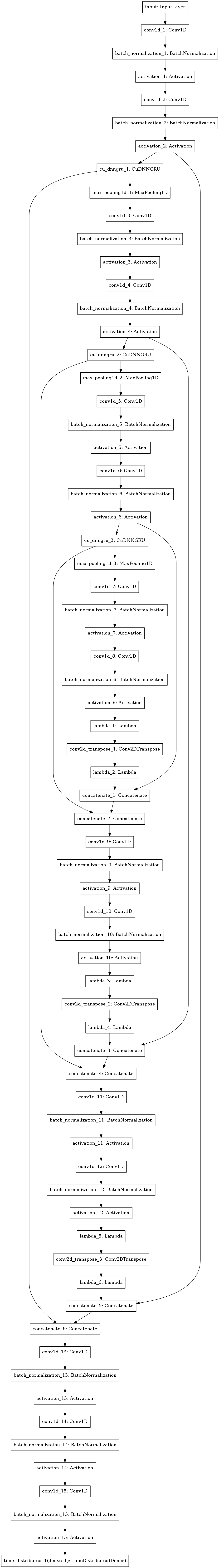

Supplement: Supplementary file 1 — Additional file 1 Figure S1. UR-net’s network structure plotted by Keras. [file 12859_2020_3459_MOESM1_ESM.png]
